# Antibiotic Treatment of the Tick Vector *Amblyomma americanum* Reduced Reproductive Fitness

**DOI:** 10.1371/journal.pone.0000405

**Published:** 2007-05-02

**Authors:** Jianmin Zhong, Algimantas Jasinskas, Alan G. Barbour

**Affiliations:** 1 Department of Microbiology and Molecular Genetics, Department of Medicine and Pacific-Southwest Center for Biodefense and Emerging Infections, University of California Irvine, Irvine, California, United States of America; 2 Department of Biological Sciences, Humboldt State University, Arcata, California, United States of America; The Scripps Research Institute, United States of America

## Abstract

**Background:**

The lone star tick *Amblyomma americanum* is a common pest and vector of infectious diseases for humans and other mammals in the southern and eastern United States. A *Coxiella* sp. bacterial endosymbiont was highly prevalent in both laboratory-reared and field-collected *A. americanum*. The *Coxiella* sp. was demonstrated in all stages of tick and in greatest densities in nymphs and adult females, while a *Rickettsia* sp. was less prevalent and in lower densities when present.

**Methodology/Principal Findings:**

We manipulated the numbers of both bacterial species in laboratory-reared *A. americanum* by injecting engorged nymphs or engorged, mated females with single doses of an antibiotic (rifampin or tetracycline) or buffer alone. Burdens of the bacteria after molting or after oviposition were estimated by quantitative polymerase chain reaction with primers and probes specific for each bacterial species or, as an internal standard, the host tick. Post-molt adult ticks that had been treated with rifampin or tetracycline had lower numbers of the *Coxiella* sp. and *Rickettsia* sp. and generally weighed less than ticks that received buffer alone. Similarly, after oviposition, females treated previously with either antibiotic had lower burdens of both bacterial species in comparison to controls. Treatment of engorged females with either antibiotic was associated with prolonged time to oviposition, lower proportions of ticks that hatched, lower proportions of viable larvae among total larvae, and lower numbers of viable larvae per tick. These fitness estimators were associated with reduced numbers of the *Coxiella* sp. but not the *Rickettsia* sp.

**Conclusion/Significance:**

The findings indicate that the *Coxiella* sp. is a primary endosymbiont, perhaps provisioning the obligately hematophagous parasites with essential nutrients. The results also suggest that antibiotics could be incorporated into an integrated pest management plan for control of these and other tick vectors of disease.

## Introduction

Endosymbiotic bacteria inhabit a variety of invertebrates [1]. Ticks, which are arachnids and a parasitic form of mite, are not an exception [2]. Bacteria classified in the genera *Francisella, Rickettsia*, or *Coxiella* have been found in both hard (ixodid) and soft (argasid) ticks [3]–[8]. These genera also include species that are pathogenic for vertebrates, including humans, and are transmitted to vertebrates by the same types of ticks. For example, ixodid ticks of the genera *Rhipicephalus* and *Haemaphysalis* and an argasid tick of the genus *Ornithodoros* [6] not only are bearers of uncharacterized bacteria of the genus *Coxiella* but also are vectors of *Coxiella burnetii*, the Q fever agent [9].

We recently identified and characterized a hitherto unknown *Coxiella* sp. in the ixodid tick *Amblyomma americanum*, also known as the lone star tick [10]. Three lines of evidence indicate that this *Coxiella* sp. is an endosymbiont of *A. americanum* [10]: (1) The organism has been found in all *A. americanum* ticks examined to date, including field-collected samples from several locations and from laboratory-reared specimens. In contrast, a *Rickettsia* sp. was not detectable in some of the same sample. (2) The *Coxiella* sp. was present in eggs, an indication of transovarial transmission. (3) The organism appears to have a reduced genome, a feature of several endosymbionts of invertebrates [11].

The finding of endosymbiotic bacteria in an arthropod that is obligately hematophagous at all stages of its life cycle led to questions about the functions, if any, of the bacteria for its host's physiology and reproduction. For instance, could the endosymbionts provide one or more essential nutrients that are absent or scant in the tick's blood meal? With this longer term goal in mind, we set out to determine first whether manipulation of the numbers of the bacteria in the ticks could discernibly affect the tick host of the bacteria. Application of antibiotics has been a useful approach for investigating the role of endosymbionts for their invertebrate hosts, for example in filarial worms and weevils [12], [13]. In some cases there is no apparent effect on the host, but in others the antibiotic treatment reduced the fitness of the animal or otherwise revealed contributions of the bacterium to the physiology of the host.

Disease prevention was another rationale for assessing the effect of antibiotics on the endosymbiotic bacteria and, indirectly, on the ticks themselves. If antibiotic treatment was associated with both reduced numbers of bacteria and lowered fitness of ticks, this would justify further investigation of the bacteria as targets for vector control efforts. We used the lone star tick as a model, in part because it is easily reared in the laboratory. But, in addition, this tick species is of growing importance as a pest and disease vector in the United States for humans and agricultural animals [14], [15]. Thus, the findings from these studies potentially could find application in an integrated pest management system for public and veterinary health benefit [16]. Because an antibiotic compound might have effects on ticks that are not attributable to its antimicrobial properties, we used two antibiotics with different mechanisms of action, tetracycline and rifampin, both of which were expected to have activity on the *Coxiella* sp. and *Rickettsia* sp. bacteria in the ticks [17]–[20].

## Materials and Methods

### Ticks

Engorged or flat *A. americanum* larvae, nymphs, and adults or egg masses were obtained in late 2005 and early 2006 from the Department of Entomology and Plant Pathology of Oklahoma State University, Stillwater, OK. The tick colony was started in 1976 with engorged females collected in Cherokee County, OK. Engorged females are introduced every 2 years in approximately equal numbers to mated colony females, and since 1991 they have been obtained from Payne County, OK. The most recent introduction of field-collected females had been in July 2004. The nymphs had fed on rabbits, and adults ticks had fed on sheep. Ticks were maintained in a Model I-36 VL Percival Scientific environmental chamber (Percival Scientific, Perry, IA) at 24°C, 94–96% humidity, and on a 12:12-hr light:dark cycle [21]. Engorged nymphs were kept individually in 20 ml polystyrene cups with cotton mesh coverings. Engorged adults and egg masses were kept in 15 ml polystyrene centrifuge tubes with glass-wool plugs and which had been tared. Tick and egg mass weights were determined on a Sartorius model CP2P microbalance with a sensitivity of 1 µg. Before experiments individual ticks were weighed and then grouped so that the average weight and standard deviation were similar for each group.

### Antibiotic treatments

Stock solutions of tetracycline HCl or rifampin (Sigma, St. Louis, MO) were made in 70% ethanol or in pharmaceutical grade dimethylsulfoxide (Sigma), respectively, at concentrations of 100 mg/ml. The antibiotics were diluted in 137 mM NaCl-2.7 mM KCl-4.3 mM sodium phosphate-1.4 mM potassium phosphate, pH 7.0 (PBS). The doses of each antibiotic were 12.5 ng per mg weight of engorged nymphs and 17 ng per mg weight of engorged, mated female adults. On their fourth day after repletion and dropping from the host mammal, engorged nymphs or females were weighed and then injected under a dissecting microscope with a locally-fabricated injection apparatus and glass capillaries that had been pulled with a Sutter needle puller. The ticks were affixed on their sides to double-sided tape adhered to a glass slide, and the slide was adjusted to a 45° angle to the microscope stage. The tip of the capillary was inserted into the hemocoel through the space between the first and second legs. After injection of 0.25 µl into nymphs or 0.5–0.8 µl into females, the needle was left place for at least 30 sec and then slowly withdrawn. For treatments of engorged nymphs, the adult ticks were sexed and weighed after molting. For experiments with engorged females, unhatched eggs, live larvae, and dead larvae were counted under a dissecting scope 7 d after oviposition was complete for a given female.

### Quantitative polymerase chain reaction (PCR) assay

Ticks were frozen and pulverized in liquid nitrogen, and then DNA was extracted as described [22], [23]. DNA concentrations were determined on a NanoDrop ND-1000 spectrophotometer (Nanodrop Technologies, Inc.). Quantitative PCR with labeled probes was carried out in a Rotor-Gene RG-3000 apparatus (Corbett Research) as described [10]. In brief, the PCR targets for the bacteria were the *fusA* (elongation factor G) gene of the *Coxiella* sp. and the *gltA* (citrate synthase) gene of the *Rickettsia* sp. [24]. The GenBank accession number for the partial *gltA* sequence of the *Rickettsia* sp. of the laboratory-reared *A. americanum* was EF514206. For estimating gene copies in *A. americanum*, we used the macrophage inhibitory factor (MIF) gene, for which we had both genomic sequence and mRNA sequence and which occurs in single copy per genome [25]. Standard curves were determined from targets cloned into an *Escherichia coli* plasmid, and these were serially diluted from 10^6^ to 10^−1^ gene copies per reaction [10]. The assays had coefficient of determinations (*R^2^*) for the log regression of the *C_t_* values vs. DNA dilutions of 0.99, efficiencies of>92%, and sensitivities of 1–10 gene copies.

### Statistical analysis

Standard parametric statistical analyses were carried out with SYSTAT v. 11 (SYSTAT Software, Inc.), and significance tests were two-sided. Ratios and skewed data were log-transformed for significance testing. Similarly, means and 95% confidence intervals (CI) were determined for log-transformed data, and then the antilogs were calculated for presentation. The Mann-Whitney-Wilcoxon and Spearman non-parametric tests were exact and carried out with StatXact v. 6.3 (Cytel Software, Inc.). For the Kaplan-Meier survival analysis we used the Mantel log-rank test statistic. Exact logistic regression (LogXact v. 6.3, Cytel Software, Inc.) was used to evaluate the association between scalar predictor variables and discrete outcomes. Box-and-whisker plots in figures show the median, first and third quartiles, and 1.5X interquartile range.

## Results

### *Coxiella* sp. and *Rickettsia* sp. by stage and age

**Figure 1:**
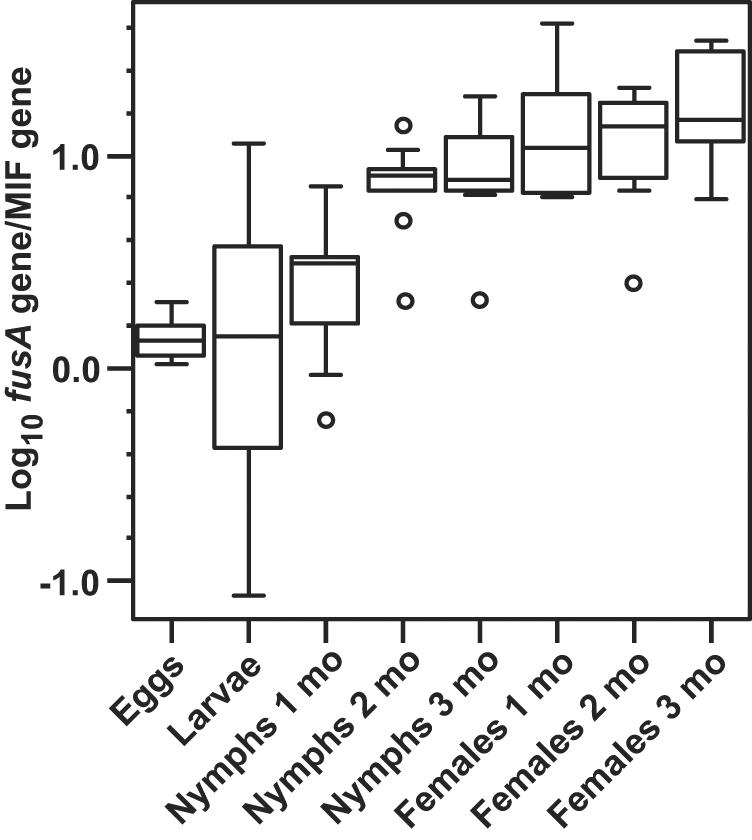
Box-and-whisker plots of log_10_ ratios of the copies of the endosymbiont *Coxiella* sp. *fusA* gene to copies of the host *Amblyomma americanum* MIF gene in different stages (larvae, nymphs, and female adults) and ages after molt of the host ticks. Gene copies per tick were estimated by quantitative polymerase chain reaction assay with gene specific primers and probes.

The ratios of the *fusA* gene of the *Coxiella* sp. to the MIF gene of the *A. americanum* in a given tick were used to estimate the burdens of the bacterium in the ticks at different stages of their life cycle. Figure 1 shows box-whisker plots of the results for egg masses, flat larvae, flat nymphs at different times after molting, and flat females at different post-molt times. The medians for the *fusA*/MIF gene ratio were similar for the eggs and larvae, but the variance of the ratios was substantially greater for the larvae. In contrast, there were an estimated ∼10 *Coxiella* sp. bacteria for every tick cell by the time nymphs were 2 months post-molt, and these levels persisted through adults 3 mo. post-molt. Moreover, with the exception of nymphs 1 mo. post-molt, the variances were comparatively lower for nymphs and adults.

The *Rickettsia* sp. was not as prevalent, and, when present, the bacterial burden per tick was lower than with the *Coxiella* sp. By the criterion of a *gltA* to MIF gene ratio of>0.001, the *Rickettsia* was not detectable in the egg masses (n = 5), 1 mo. nymphs (n = 10), or 3 mo. nymphs (n = 7). Six of 20 larvae had detectable *Rickettsia* sp., but the median *gltA*/MIF ratio among positive ticks was only 0.002. Of the 10 nymphs 2 mo. post-molt, only 1 had detectable *Rickettsia*. For the 1 mo., 2 mo., and 3 mo. post-molt females the prevalences of the *Rickettsia* sp. were 7 of 10, 7 of 10, and 6 of 10, respectively. Among the positive ticks, the median numbers of *Rickettsia* sp. bacteria per tick cell increased to 0.44 and 0.27 for 1 mo. and 2 mo. females, respectively, but declined to 0.002 in 3 mo. females.

Among 11 male adult ticks that were studied, all had the *Coxiella* sp., but, as expected from the studies of post-molt females, only 7 of 11 males had detectable *Rickettsia* sp. Male ticks had lower densities of the *Coxiella* sp. than did adult females; for the *fusA*/MIF ratio the median was 0.73 in males, and the mean (95% confidence interval [CI]) was 0.76 (0.39–1.13). In contrast the density of the *Rickettsia* sp. in infected males was about the same as found in infected post-molt females; for the *gltA*/MIF ratio the median was 0.68, and the mean was 0.71 (0.53–0.89). We dissected 4 adult females and determined that the mean *fusA*/MIF ratio was 381 (252–512) in the midgut and 79 (39–298) in the ovaries, but only 12 (11–14) in the salivary glands.

These findings were further evidence that the *Rickettsia* sp. was not as prevalent as the *Coxiella* sp. in the ticks at any stage, and among ticks with the *Rickettsia* sp. the burdens of this bacterium were considerably lower than the *Coxiella* sp.

### Antibiotic treatment of engorged nymphs

In the first experiment nymphs were injected with PBS alone or PBS with tetracycline. After the molt, the means (95% CI) for the *fusA*/MIF gene ratios for the *Coxiella* sp. were 4.0 (2.7–6.0) for 7 adult females who received PBS alone and 1.2 (0.5–2.9) for 8 females who received tetracycline (*t* test *p* = 0.02; exact Mann-Whitney-Wilcoxon *p* = 0.02). The post-molt weights were 4.2 (3.9–4.4) mg for the PBS control group and 3.8 (3.6–4.0) mg for the tetracycline group (*t* test *p* = 0.06; exact Mann-Whitney-Wilcoxon *p* = 0.03).

In the second, larger experiment engorged nymphs were divided into three groups of 30 each and injected with PBS alone, PBS with rifampin, or PBS with tetracycline (day 0). The mean weights (and standard deviations) for pre-treatment nymphs were similar at 8.6 (2.2), 8.6 (2.2), and 8.5 (2.2) mg for the PBS, rifampin, and tetracycline groups, respectively. The ticks began molting by day 23; molting was complete by day 27. There was not a difference between the three groups in the onset of the molt or its last day. The 19 females in each of the PBS and tetracycline groups and the 17 females in the rifampin group were further studied. The means (95% CI) for the *fusA*/MIF gene ratios for the *Coxiella* sp. in post-molt adults were 9.3 (5.8–12.9) for the PBS control, 0.9 (0.3–1.6) for rifampin (*t* test *p*<0.001), and 1.2 (0.6–1.8) for tetracycline (*p*<0.001). Corresponding values for the *gltA*/MIF gene ratios for the *Rickettsia* sp. were 4.3 (1.5–7.0) for the PBS control, 1.8 (1.4–2.1) for rifampin treatment (*p*<0.01), and 1.4 (1.1–1.7) for tetracycline treatment (*p*<0.001). The mean post-molt weights were 5.6 (5.4–5.9) mg for PBS, 5.1 (4.8–5.5) mg for rifampin (*p*<0.01), and 4.7 (4.4–4.9) mg for tetracycline (*p*<0.01).

### Effect of antibiotics on reproductive fitness

The study of bacterial burdens by stage showed that overall densities of the *Coxiella* endosymbiont in *A. americanum* were lowest in eggs and rose in number through the nymph stage and remained stable as the tick matured as an adult, suggesting that the greatest impact of symbiont manipulation would be on reproduction. To investigate this we injected 30 fed, mated females in groups of 10 with mean weights of 0.6 g with PBS, rifampin, or tetracycline on day 0. The ticks were maintained individually in an incubation chamber and observed daily for oviposition. The date for the start of oviposition was noted, and marks were made daily on the tube to monitor oviposition progress as the female advanced along the tube. When oviposition ceased, the female was removed, the tubes were weighed, and the tube's tare was subtracted to estimate the egg mass. The egg masses in the tubes were returned to the same incubation conditions, and the start date for hatching of the eggs was recorded. Seven days after hatching commenced, the number of viable larvae, dead larvae, and unhatched eggs were counted in each tube.

**Figure 2:**
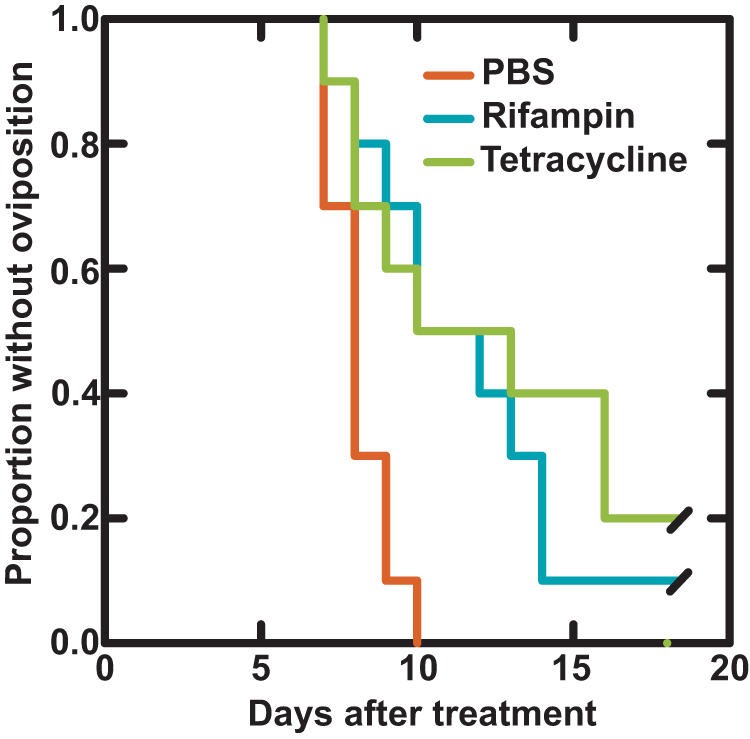
Proportion of *A. americanum* females without oviposition by day after treatment by injection with single doses of phosphate-buffer saline (PBS; n = 9), rifampin (n = 10), or tetracycline (n = 10). Time was censored from day 18.

One of the 10 control ticks started oviposition before day 10 but died before its oviposition was complete, and this tick was excluded from subsequent analysis. Figure 2 shows the proportion of ticks that had not commenced oviposition by day of observation. For survival analysis, day 18 was the censored time for ticks that had not started oviposition by that day. The time to oviposition was delayed for antibiotic-treated ticks (Chi square 11.3, 2 degrees of freedom; *p* = 0.004).

**Figure 3:**
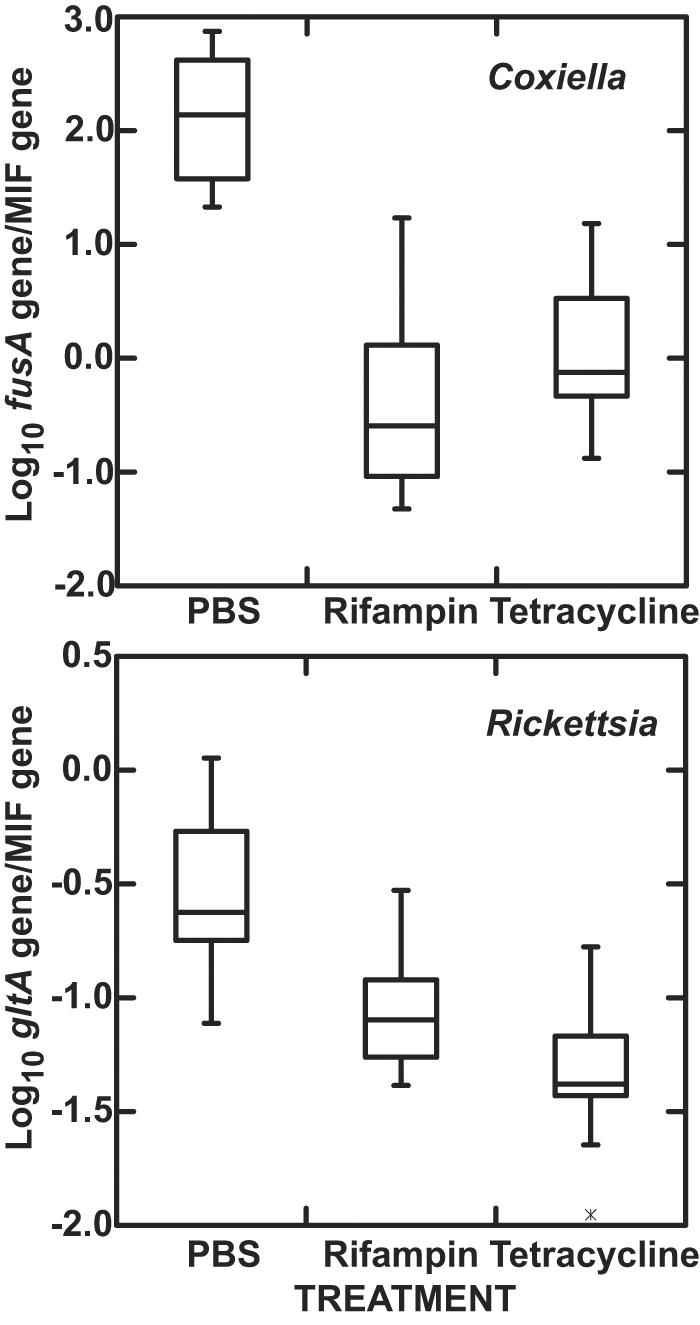
Box-and-whisker plots of log_10_ ratios of *fusA* gene of *Coxiella* sp. to MIF gene of *A. americanum* (upper panel) or log_10_ ratios of *gltA* gene of *Rickettsia* sp. to MIF gene (lower panel) in post-oviposition ticks that were treated by injection with single doses of PBS (n = 9), rifampin (n = 10), or tetracycline (n = 10) as fed, mated females.

Figure 3 shows the total burdens of the *Coxiella* sp. and the *Rickettsia* sp. in the females after oviposition. In the control group the ratio of the *Coxiella* sp. *fusA* gene to the MIF gene was approximately 10-fold higher than in flat female adults (Figure 1). In contrast, the estimated numbers of the *Coxiella* sp. were ∼100-fold lower in the both the rifampin-and tetracycline-treated females. The overall *Rickettsia* sp. burden per tick was substantially lower than the *Coxiella* sp. in the control group, and the effect of antibiotic treatment was less marked on the *Rickettsia* sp. than on the *Coxiella* sp.

Table 1 summarizes indicators of the reproductive fitness in each experimental group and their associations with antibiotic treatment. The antibiotic-treated ticks had ratios of egg mass weights to pre-oviposition weights that tended to be lower than those of PBS-injected ticks. More pronounced differences between antibiotic and control groups were observed for the other variables. Rifampin and tetracycline treatments were associated with a significantly lower proportion of ticks that hatched, a lower proportion of viable larvae among total larvae that hatched, and lower number of viable larvae per tick.

**Table 1:** Effect of antibiotic treatment of *A. americanum* females on their eggs and offspring

Variable	PBS (95% CI)*	Rifampin (95% CI)	Tetracycline (95% CI)
Number of ovipositing ticks/total number of ticks	9/9	9/10	8/10
Mean egg mass weight/pre-oviposition female weight	0.38 (0.33–0.44)	0.32 (0.26–0.39)	0.26† (0.17–0.38)
Mean proportion of a tick's eggs that hatched	0.96 (0.90–1.0)	0.11^§^ (0.0–0.25)	0.48^§^ (0.24–0.62)
Mean proportion of a tick's hatched larvae viable at 7 d	0.96 (0.94–0.98)	0.12^§^ (0.01–0.23)	0.48^§^ (0.25–0.71)
Mean number of viable larvae per ovipositing tick	1695 (1153–2492)	4^§^ (0–19)	81^§^ (13–489)

* 95% confidence interval; †*t* test *p* value<0.1 for antibiotic treatment vs. PBS control; ^§^ *t* test *p* value<0.01 for antibiotic treatment vs. PBS control

We used a binary fitness index where “0” was assigned to a tick with no eggs or a ratio live larvae to dead larvae plus unhatched eggs ≤ 1.0, and “1” was assigned to a tick with a ratio of live to dead larvae plus unhatched eggs of>1.0. Using logistic regression, there was no association between the fitness index and the *Rickettsia* sp. *gltA*/MIF gene ratio (odds ratio [OR] of 1.4, 95% CI of 0.6–3.2; *p* = 0.4), but there was an association between the fitness index with the *fusA*/MIF ratio (OR 8470, 6.9-∼10^6^; *p* = 0.008) in the post-oviposition female. By the same measure, there was no association between the pre-treatment weight of the female and the fitness index (OR 18.5, 0-∼10^4^, *p* = 0.55). We also examined the relationship between the ratio of live larvae to dead larvae plus unhatched eggs to estimated burdens of the *Coxiella* sp. and the *Rickettsia* sp. by Spearman rank correlation. There was an association (*R^2^* = 0.36; *p* = 0.002) between a higher viable larvae ratio and lower *Coxiella* sp. burdens but not between the viable larvae ratio and *Rickettsia* sp. burdens (*R^2^* = 0.08; *p* = 0.75).

**Figure 4:**
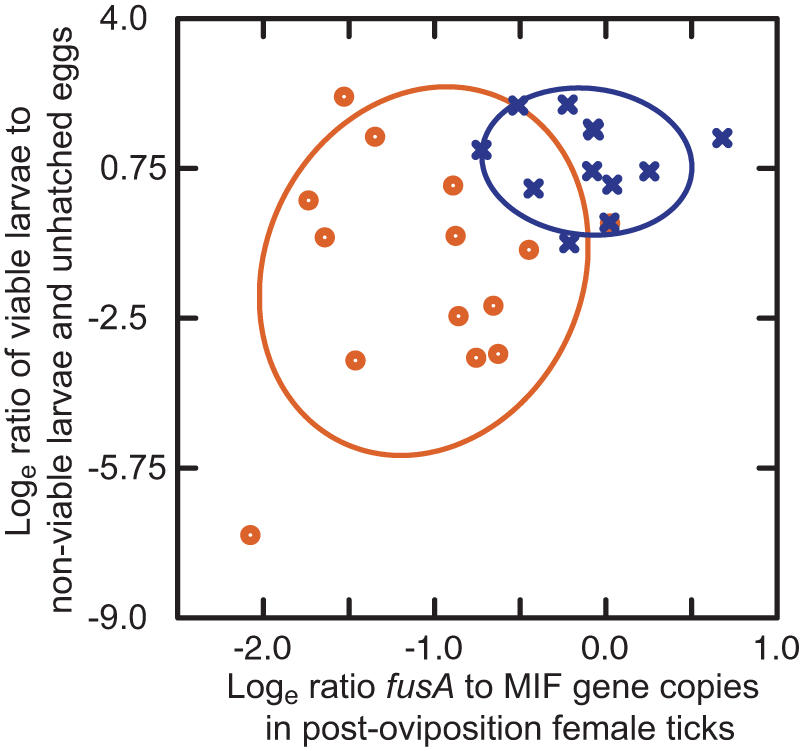
Association between post-oviposition burdens of the *Coxiella* sp., as estimated by *fusA*/MIF gene ratios, and reproductive fitness, as estimated by the ratio of viable larvae to non-viable larvae and unhatched eggs, in female *A. americanum* after injection of PBS (blue X) or rifampin (red circle). The ratios have been log-transformed. The blue and red ellipses correspond to bivariate standard deviations of the means of the *x* and *y* variables for each treatment group, respectively.

Although the final concentration of the intial diluent, dimethylsulfoxide (DMSO), in the rifampin solution for injection was only 10% (v/v) and less than 0.08 µl DMSO was injected, it was possible that this substance could account wholly or in part for the observed effects of rifampin. Therefore, we carried out another experiment in which engorged, mated females were injected with 1 µl PBS/10% DMSO (n = 12) or 1 µl PBS/10% DMSO with 10 µg rifampin (n = 14). Figure 4 plots an estimate of reproductive fitness for each female tick against the burdens of the *Coxiella* sp. in the female after oviposition. The mean *fusA* to MIF gene ratio was 2.8 (2.2–3.5) for the control group and 1.5 (1.3–1.7) in the rifampin-treated group (*t* test *p* = 0.0001). The mean ratio (95% CI) of live larvae to dead larvae plus unhatched eggs was 2.5 (1.5–4.4) for the control group and 0.25 (0.07–0.88) for the rifampin-treated group (*p* = 0.005). Thus, the lowered reproductive fitness observed again in this second experiment could be not be attributed to the DMSO.

## Discussion

In this series of experiments we further characterized two bacterial species that occur in both laboratory-reared and field-collected *A. americanum* ticks [10]. We first confirmed that *Coxiella* sp. was highly prevalent in all sampled ticks and then documented that it was present in all stages, reaching its highest densities in older nymphs and in adults, as assessed by species-specific quantitative PCR of whole tick extracts with a tick gene as an internal standard. In contrast, the *Rickettsia* sp. was less prevalent among the ticks, and, when present, in significantly lower densities than the *Coxiella* sp. While we cannot conclusively identify the *Rickettsia* sp. in this study, it is probably synonymous to what has been named “*Rickettsia amblyommi*” [26]. The partial sequence of the citrate synthase gene of the *Rickettsia* sp. in our study was identical to the *gltA* sequence of “*R. amblyommi*” from *Amblyomma* spp. ticks in Brazil and Argentina (AY375163 and DQ517290), as well as the *gltA* sequences of two unnamed *Rickettsia* sp. (AF031496 and AY3888929) from *A. americanum*. Moreover, Mixson et al. reported that “*R. amblyommi*” was transovarially transmitted and had a prevalence in *A. americanum* that was similar to what we found in our present and previous studies [27].

It is possible another bacterial species besides the *Coxiella* and *Rickettsia* species was present in *A. americanum* and vertically transmitted. But if a third (or fourth) species was present, unlike the *Coxiella* and *Rickettsia* species, it was not represented in a large cDNA library of *A. americanum* obtained from the same tick rearing facility [10]. Some other ixodid ticks, such as *Dermacentor*, have a *Francisella*-like endosymbiont, but these endosymbionts have not been found in *Amblyomma* species [8]. A few of the females introduced into the colony in mid-2004 may have been infected with *Borrelia lonestari*, which has a prevalence of about 1–2% across the south-central and southeastern regions of the U.S. [28]. But if *B. lonestari* organisms were originally present in one or more of the introduced females in 2004, they likely would have been lost or further diluted during subsequent generations in the laboratory facility during the ensuing year. In any case, *Borrelia* spp. are resistant to rifampin [29], [30], so one could not attribute rifampin's effects on fitness to its inhibition of spirochetes. Finally, *Ehrlichia* pathogens may have been present in a few of the field-collected ticks that were introduced into the colony in 2004. But *E. chaffeensis* is not transmitted transovarially in *A. americanum* [31], and, thus, would not be expected in the laboratory-reared ticks we used.

From the descriptive study of the *Coxiella* and *Rickettsia* species, we proceeded to what, to our knowledge, is the first attempt at manipulation of endosymbiont numbers in ticks. For this aim we administered single injections of either of two antibiotics or buffer alone to engorged nymphs or engorged, mated females. Ticks that received tetracycline or rifampin as nymphs generally weighed less as adults than ticks that had received buffer alone. This effect was associated with lower numbers of both the *Coxiella* sp. and the *Rickettsia* sp. in post-molt ticks. When examined with estimators of fitness, treatment of engorged females with either of these antibiotics was associated with prolonged time to oviposition, lower proportions of ticks that hatched, lower proportions of viable larvae among total larvae that hatched, and lower numbers of viable larvae per tick.

An association between symbionts and reproductive success has also been noted in other arthropods. An early demonstration was Harington's experiments in which he prevented the triatomine *Rhodnius prolixus* from acquiring its bacterial symbiont, *Rhodococcus rhodnii*, and demonstrated lower fecundity in the symbiont-free insects [32]. Other examples include the increased fecundity and reproductive fitness associated with endosymbionts that has been reported for predatory mites infected with a Cytophaga-like intracellular bacterium [33], tsetse flies infected with *Wigglesworthia glossinidia* [34], and carpenter ants infected with *Blochmannia floridanus* [35].

For this study we used two antibiotics: tetracycline and rifampin. Both antibiotics inhibit the growth of *Coxiella burnetii* in tissue culture cells at minimum inhibitory concentrations (MIC) of 1 to 4 µg/ml [17], [18] and of *Rickettsia* species in similar concentrations [36], [37]. On a total weight basis and with the assumption of free distribution of the antibiotic after injection, the doses used in this study would provide for concentrations of 10–20 µg/ml in a tick. Both tetracycline and rifampin have also been successfully used to cure or lower the numbers of endosymbionts in other arthropods, including mosquito vectors of disease [38]–[41]. In a report on the endosymbiont *Wolbachia pipientis* of mosquitoes and growing in tissue culture, tetracycline and rifampin (MIC 0.125 µg/ml) were each more effective than fluoroquinolones, which had MICs of 1–4 µg/ml, or macrolides, which had MICs of 8–64 µg/ml [19].

Both antibiotics inhibit protein synthesis, either by interference with DNA-dependent RNA polymerase in the case of rifampin or at the level of translation by tetracycline. For many bacteria these antibiotics are bacteriostatic rather than bactericidal. Therefore, the antibiotics may have affected endosymbiont function without necessarily reducing the number of bacteria. The results in the second experiment with adult females and summarized Figure 4 provides evidence in support of this: the reproductive fitness of some females was low even when their *Coxiella* sp. burdens were not lower than those of the control group following oviposition.

While we cannot rule out a contribution of the *Rickettsia* sp. to the effects observed in this study, the evidence to date indicates a greater or exclusive role of the *Coxiella* sp. The absence of detectable *Rickettsia* sp. DNA in ∼30% of *A. americanum* in field-collected samples as well as in a laboratory colony of ticks suggests that the *Rickettsia* sp. is not essential for tick viability or reproduction. When the *Rickettsia* sp. was also present in ticks, along with the *Coxiella* sp., it was in lower densities than the *Coxiella* sp. and declined in numbers as adults aged. Although both the *Coxiella* sp. and *Rickettsia* sp. had lower numbers in antibiotic treated ticks, a direct correlation between bacterial densities and measures of reproductive fitness were observed for the *Coxiella* sp. but not the *Rickettsia* sp.

Since the experiment was carried out in the laboratory and with ticks from a long-established breeding colony, we cannot ascribe the effects of antibiotics on fitness to increasing the vulnerability of endosymbiont-free ticks to parasites, such was the case with pea aphids (*Acyrthosiphon psium*), an endosymbiont of the aphid, and the hymenopteran parasitoid, *Aphidius ervi*, a major natural enemy of aphids [42]. Instead, we propose that the apparently obligate association between *A. americanum* and the *Coxiella* sp., is based on nutritient provisioning by the endosymbiont [43], as has been demonstrated with endosymbionts of aphids [44] and a bacterial endosymbiont in the trypanosomatid *Herpetomonas roitmani* [45]. Ticks are obligately hematophagous, and the blood of their hosts may have insufficient concentrations of what for the tick are essential nutrients, such as amino acids, nucleosides, vitamins, and other enzyme co-factors. If ticks lacked those nutrients during periods of higher biosynthetic demands, such as during the molt and reproduction, this could manifest, depending on when the antibiotic was administered, as lower weights of adults and lowered fecundity and total numbers of surviving larvae. An antibiotic need not kill the bacteria to have an effect at these critical periods in tick development and reproduction; it need only inhibit transcription or translation by the symbiont.

If administration of antibiotics to ticks, *A. americanum* or another vector, reduced their fitness or otherwise impaired their competence as infection vectors, this would offer a tool not only for vector biology and ecology research but also for control of tick as disease vectors and pests. We are mindful of the possible pitfalls of and impediments to the application of antibiotics in the environment. These include regulatory hurdles for implementation of such measures. Nevertheless, we point out that a targeted application of antibiotics as a component of an integrated pest management program may be preferable to pesticides, which unlike most antibacterial antibiotics often target enzymes or pathways that humans and other vertebrates share with arthropods and, consequently, are potentially more toxic than most antibiotics. The antibiotic could be one that is seldom if ever used as therapy in either human or veterinary medicine, but the development of cross-resistance against antibiotics of the same class could reduce its effectiveness or acceptance over time. An alternative then is development of novel antimicrobial compounds that target bacterial endosymbionts of arthropod pests and vectors but whose application is limited to that use.

In experiments in which engorged nymphs were administered an antibiotic or buffer alone, the treated ticks generally weighed less as adults than those that received buffer alone. While the consequences of weight differences between adults on their reproductive fitness were not assessed, it is possible that a persistent effect of antibiotic treatment would manifest as reduced fecundity and fewer viable offspring. In that event, antibiotics that were directed to the vertebrate hosts for nymphal ticks could affect not only the fitness of the ticks but also bacterial pathogens that had these characteristics: (a) the bacteria are transmitted to humans by the adult stage of that tick, (b) the nymphs' vertebrate hosts are reservoirs of the zoonosis, and (c) the bacteria are susceptible to that antibiotic. For example, an oral antibiotic that was administered in bait to a rodent infected with a given pathogen and at the time of nymphal ticks' greatest feeding activity could possibly result in the curing of the rodent of the infection, prevention of transmission of the pathogen to the larval tick, and fewer ticks in the next generation in that environment.
